# SUV39H1 regulates corneal epithelial wound healing via H3K9me3-mediated repression of p27

**DOI:** 10.1186/s40662-022-00275-5

**Published:** 2022-02-01

**Authors:** Shuai Yang, Weiwei Chen, Shanshan Jin, Guangying Luo, Xia Jing, Qi Liu, Peter S. Reinach, Jia Qu, Dongsheng Yan

**Affiliations:** 1 School of Ophthalmology and Optometry, Eye Hospital, Wenzhou Medical University, 270 Xueyuan Road, Wenzhou, 325027 Zhejiang China; 2 State Key Laboratory of Ophthalmology, Optometry and Visual Science, Wenzhou, Zhejiang China

**Keywords:** SUV39H1, Histone methylation, Corneal epithelial wound healing, p27, Cell proliferation

## Abstract

**Background:**

Corneal epithelial wound healing (CEWH) is vital for maintaining the integrity and barrier function of the cornea. Although histone modifications mediating gene expression patterns is fundamental in some other tissues, it remains unclear whether these gene regulation patterns underlie CEWH. Suppressor of variegation 3-9 homolog 1 (SUV39H1) plays a vital role in mediating gene silencing via histone H3 trimethylation of lysine 9 (H3K9me3). This study aims to characterize the comprehensive signature of epigenetic modifiers and determine the role of SUV39H1 in CEWH.

**Methods:**

NanoString nCounter technology was used to detect the differentially expressed epigenetic modifiers during CEWH. Bioinformatic analyses were performed to reveal their involvement in this process. After knockdown of SUV39H1 with siRNA transfection, we determined the function of SUV39H1 on cell proliferation and migration in human corneal epithelial cells (HCECs) via MTS, EdU, and wound-healing assay, respectively. Flow cytometry analysis further confirmed the effect of SUV39H1 on the cell cycle of HCECs. Loss-of-function assays for SUV39H1 with siRNA injection or chaetocin assessed the role of SUV39H1 on CEWH in vivo. Quantitative reverse transcription polymerase chain reaction (RT-qPCR) and Western blotting characterized the expression of SUV39H1 and its target genes. Chromatin immunoprecipitation assay was used to evaluate the distributions of H3K9me3 marks at the promoters of SUV39H1 target genes.

**Results:**

We first identified 92 differentially expressed epigenetic modifiers and revealed their involvement during CEWH. SUV39H1 was confirmed to be upregulated in response to corneal injury. Its downregulation significantly inhibited HCEC proliferation and retarded in vivo CEWH. Furthermore, knockdown of SUV39H1 upregulated the p27 expression level and reduced H3K9me3 marks at p27 promoter in HCECs. In addition, p27 was remarkably downregulated with elevated H3K9me3 marks at its promoter during in vivo CEWH.

**Conclusions:**

SUV39H1 plays a critical role in regulating corneal epithelial cell proliferation via H3K9me3-mediated suppression of p27 during CEWH. Our findings suggest that epigenetic modifiers such as SUV39H1 can be potential therapeutic approaches to accelerate corneal repair.

**Supplementary Information:**

The online version contains supplementary material available at 10.1186/s40662-022-00275-5.

## Background

Corneal structural integrity and transparency maintenance are both critical for preserving normal vision. The outermost epithelial layer of the cornea contributes to this process by providing a tight functional barrier against pathogenic stromal infiltration. Injury-induced declines in epithelial integrity compromise its barrier function, which renders the cornea susceptible to pathogenic infiltration, neovascularization, ulceration, and scarification [1, 2]. Corneal epithelial wound healing (CEWH) is a complex and multifactorial tissue rebuilding process that depends on cell proliferation, migration, differentiation, and stratification [3]. These orchestrated cellular processes are, in part, controlled by numerous time-dependent modulation of selective gene activation and repression, such as integrins, growth factors, and cytokines [3–5]. Thus, it is crucial to clarify how the differential gene expression is regulated and coordinated.

Epigenetic regulations convey the heritability of gene expression patterns without altering DNA sequences [6], including DNA methylation, histone modifications, and non-coding RNAs (ncRNAs). These epigenetic mechanisms are universal and efficient approaches to regulate gene expression during development and diseases [7–9]. Our recent research revealed that DNA methylation regulates CEWH by targeting miR-200a and CDKN2B [10]. An important indication of histone modification involvement in corneal repair was when suberoylanilide hydroxamic acid (SAHA) showed an inhibitory effect on corneal neovascularization via repressing histone deacetylase-mediated hemangiogenesis and inflammation pathways [11]. Furthermore, deficiency of a histone deacetylase Sirt6 delayed corneal epithelial wound closure which resulted in excessive inflammation in the corneal stroma [12]. MicroRNA (miRNA) is another epigenetic modulator whose changes in expression levels affect CEWH in mice [13]. It is noteworthy that these epigenetic mechanisms are implemented by various epigenetic modifiers [14–17]. However, little is known about whether histone modifiers contribute to regulating CEWH.

The suppressor of variegation 3-9 homolog 1 (SUV39H1) is a critical histone methyltransferase that catalyzes histone H3 lysine 9 trimethylation (H3K9me3) formation [18]. H3K9me3 is a prominent determinant of heterochromatin formation and is a hallmark of transcriptionally silent genes [19, 20]. As the homologous protein of SUV39H1, SUV39H2 shares 59% identity with it, has similar structure and enzyme activity in mice, and displays testis-specific expression [21]. The extent of gene expression suppression by SUV39H1 is dependent on its catalytic activity in mediating H3K9me3 [22–24]. For example, loss of SUV39H1 leads to an increase of E-cadherin expression by decreasing H3K9me3 marks at its promoter and ultimately inhibits breast cancer cell migration [25]. In addition, parafibromin recruits SUV39H1 to catalyze H3K9me3 marks at the cyclin D1 promoter and decreases its expression which restrains cell proliferation [26]. Interestingly, SUV39H1 and CTIP2 cooperate to silence p21 gene transcription through increasing H3K9me3 at its promoter, which accelerates cell proliferation [27]. Besides, SUV39H1 represses MyoD-dependent muscle gene myogenin expression, which inhibits differentiation of proliferating muscle cells [23]. All these findings indicate that SUV39H1 plays critical roles in controlling diverse biological processes, including cell migration, proliferation, and differentiation via H3K9me3-mediated transcriptional repression. However, it is unclear whether such a regulatory mechanism affects CEWH.

We show here that corneal epithelial injury activates SUV39H1 expression. Loss of SUV39H1 function remarkably inhibits HCEC proliferation by perturbing cell cycle processes in vitro and delays CEWH in vivo. Mechanistically, SUV39H1 upregulation accompanies the cyclin-dependent kinase inhibitor (CDKI) p27 downregulation resulting from a gain of H3K9me3 marks at its promoter during CEWH. Conversely, knockdown of SUV39H1 increases p27 expression level in human corneal epithelial cells (HCECs). This increased p27 expression is attributable to the reduction of H3K9me3 marks at its promoter. Collectively, our findings highlight the importance of SUV39H1 during CEWH and suggest it can be a potential candidate for hastening CEWH.

## Methods

### Corneal epithelial wound healing model

As previously described [13], eight-week-old C57BL/6 mice were anesthetized by intraperitoneal (i.p.) injection of xylazine (13 mg/kg) in combination with ketamine (87 mg/kg). Under a stereomicroscope, we used Algerbrush II (The Alger Company Inc., Lago Vista, TX, USA) to debride the corneal epithelium of the right eyes, which were referred to the wound healing group. The uninjured left eyes were served as the normal control group. The corneal epithelial layers were collected for RNA and protein extraction from these two different groups when the unrepaired area was approximately 10% of its initial area.

All animal treatments were performed in strict accordance with the ARVO Statement for the Use of Animals in Ophthalmic and Vision Research, and approval from Wenzhou Medical University Animal Care and Use Committee.

### NanoString nCounter assay and bioinformatic analyses

A customized mouse epigenetic regulator assay kit (nCounter v1.3; NanoString Technologies Inc., Seattle, WA, USA) was chosen based on current descriptions of epigenetic regulatory mechanisms. It contains 336 epigenetic modifiers involved in the control of DNA methylation, histone modifications, chromatin remodeling, microRNA biogenesis, and 14 housekeeping genes (Additional file 10: Table S1). Six CEWH mice were used for NanoString nCounter analysis. Total RNA (100 ng) extracted from each mouse corneal epithelium were used to establish digital epigenetic modifier profiling in accordance with the manufacturer’s instructions and the previous study [28]. Expression patterns of epigenetic modifiers were analyzed as previously described [13]. The Cluster 3.0 (Eisen Lab, University of California at Berkeley, CA, USA) was used to perform Hierarchical Clustering and the results were visualized in a clustered heat map using TreeView (Eisen Lab, University of California at Berkeley, CA, USA). The differentially expressed epigenetic modifiers were filtered with a limma algorithm [29], and selected following these criteria: (1) *P* < 0.05, false discovery rate (FDR) < 0.05; (2) fold change > 1.5 or < 0.5. Subsequently, data were clustered in terms of Euclidian distance and *Z*-score was used to normalize each row. Furthermore, Gene Ontology (GO) and Kyoto Encyclopedia of Genes and Genomes (KEGG) pathway analyses were performed on the differentially expressed epigenetic modifiers.

### Quantitative reverse transcription polymerase chain reaction (RT-qPCR)

Total RNA was isolated from freshly harvested corneal epithelium or cultured HCECs using TRIzol Reagent (Invitrogen, Carlsbad, CA, USA). Complementary DNA (cDNA) was then produced using the Reverse Transcription Kit (Promega Corp., Madison, WI, USA). RT-qPCR was carried out using Power SYBR Green PCR Master Mix (Thermo Fisher Scientific, Waltham, MA, USA) on the 7500 Fast Real-Time PCR System (Thermo Fisher Scientific). β-actin or GAPDH was used as the endogenous control to normalize the relative expression of target genes, which were further analyzed and expressed based on the 2^−ΔΔCt^ method (see Additional file 11: Table S2 for primer sequences).

### Western blotting analysis

RIPA lysis buffer (Beyotime Biotechnology Inc., Shanghai, China) was used for total protein extraction from freshly harvested corneal epithelium or cultured HCECs, and total protein concentration was determined with the Pierce™ Rapid Gold BCA Protein Assay Kit (Thermo Fisher Scientific). Western blotting was performed as previously described [13]. Antibodies for H3K9me3 (Diagenode Inc., pAb-056-050, Denville, NJ, USA), p27 (Cell Signaling Technology, 3686, Beverly, MA, USA), p-Rb (Cell Signaling Technology, 8516), β-actin (Cell Signaling Technology, 3700), GAPDH (Cell Signaling Technology, 5174), SUV39H1 (Merck Millipore, 07-550, Billerica, MA, USA), p21 (Merck Millipore, 05-345), and SUV39H2 (Abcam, ab190870, Cambridge, MA, USA) were used to label their target proteins. Protein bands were analyzed using ImageJ software (1.46r, Wayne Rasband, National Institutes of Health, Bethesda, MD, USA).

### Cell culture and siRNA transfection

HCECs were cultured in DMEM/F12 medium (Invitrogen) containing 10% fetal bovine serum (Invitrogen) at 37℃ with 5% CO_2_, as previously reported [30]. Depending on the experimental requirements, different amounts of HCECs were seeded onto appropriate sizes of tissue culture containers. SUV39H1 and/or SUV39H2 siRNA (50 nM, Sigma-Aldrich, St Louis, MO, USA) or negative control (NC) were transfected into HCECs using Lipofectamine RNAiMAX (Invitrogen).

### Cell proliferation assay

3 × 10^3^ HCECs were seeded onto each designated well of 96-well plate (Corning Inc., Corning, NY, USA) for the transfection of 50 nM SUV39H1 and/or SUV39H2 siRNA or NC. After 24-h transfection, the MTS assay kit (Promega) was used to quantify cell proliferation according to the manufacturer's instructions. In addition, HCEC proliferation was determined by using the Click-iT™ EdU Alexa Fluor 594 Imaging Kit (Invitrogen) as previously described [10]. The fluorescence intensity was detected and analyzed via MD ImageXpress Micro (Molecular Devices, San Jose, CA, USA).

### Double thymidine block (DTB) and cell cycle distribution analysis

HCECs were synchronized in the G1-phase using double thymidine block as published previously [31, 32]. 4 × 10^5^ HCECs were seeded onto a six-well plate (Corning) and transfected with 50 nM SUV39H1 and/or SUV39H2 siRNA or NC. Transfected HCECs were cultured in DMEM/F12 medium containing 2 mmol/L thymidine (Sigma-Aldrich) for 16 h for the first blocking. Then, HCECs were cultured in normal DMEM/F12 medium without thymidine for 9 h after washing twice with PBS. Subsequently, HCECs were treated with 2 mmol/L thymidine for another 16 h for the second blocking. Afterward, the double-blocked HCECs were released by washing twice with PBS and then incubated in thymidine-free medium. The synchronized HCECs were collected to stain with propidium iodide (PI) and analyzed for DNA content with a fluorescence activated cell sorting (FACS) caliber (Becton Dickinson) at 0 h, 6 h, 8 h, 10 h, 12 h, 14 h, 16 h, 18 h, and 24 h after release from the DTB.

### In vitro scratch wound assay

1.2 × 10^5^ HCECs were plated onto a 12-well plate (Corning) and transfected with either 50 nM SUV39H1 siRNA and/or SUV39H2 siRNA or NC at approximately 30% confluence. After 48 h, HCECs were wounded with a 100 μL pipette tip and cultured in 1 ml fresh serum-free DMEM/F12 medium. Cell migration was monitored based on photographs of remaining open areas in the cell cultures (Imager Z1; Zeiss, Jena, Germany) and quantified at 0 h and 24 h after scratching with ImageJ software. The initially injured area was designated as S_0h_, and the remaining injured area at 24 h after scratching was referred to as S_24h_. Accordingly, percentage of cell migration = (S_0h _− S_24h_)/S_0h_ × 100%.

### Flow cytometry analysis of p-Rb and CDK6 in HCECs

4 × 10^5^ HCECs were seeded onto a six-well plate and transfected with 50 nM SUV39H1 or NC. Transfected HCECs were collected to perform FACS with Intracellular Flow Cytometry Kit (Cell Signaling Technology) according to the manufacturer's protocol. Briefly, HCECs were fixed with 4% formaldehyde for 15 min at room temperature. After washing with PBS, fixed HCECs were stored at − 20 °C in 90% methanol overnight. Then, HCECs were incubated with antibodies of phospho-Rb (Alexa Fluor® 488 Conjugate, 4277, Cell Signaling Technology) or CDK6 (Alexa Fluor® 488 Conjugate, ab198944, Abcam) or IgG (Alexa Fluor® 488 Conjugate, ab150077, Cell Signaling Technology) for 1 h. Furthermore, HCECs were resuspended in PBS after washing and analyzed with the Accuri™ C6 Plus (Becton Dickinson). The FlowJo software (Tree Star Inc., Ashland, OR, USA) was used to analyze the data.

### Loss-of-function assays for SUV39H1 in vivo

Loss-of-function of SUV39H1 was performed by inhibiting its enzymatic activity with chaetocin (Selleck Chemicals Inc., Houston, TX, USA) or gene silencing via corneal intrastromal SUV39H1 siRNA injection. Here, we established CEWH models by debriding the corneal epithelium with an Algerbrush II under a stereomicroscope after demarcating a 2-mm wide corneal epithelial area with a 2-mm trephine in both right and left eyes. After creating the wound, 50 μM chaetocin was instilled into the right eyes every 2 h, whereas the contralateral control group eyes were treated with physiological saline at the same time intervals. In addition, SUV39H1 gene silencing was carried out following an established method for siRNA delivery into the corneal epithelial layer [10, 33]. We used a 33-gauge needle (Hamilton, Bonaduz, Switzerland) to inject SUV39H1 siRNA (100 μM) or NC (100 μM) in a complex with polyethylenimine (PEI; Polyplus-transfection, New York, NY, USA) into the anterior stroma of the corneas under a stereomicroscope. The right eyes were injected with SUV39H1 siRNA as the experimental group; meanwhile, the left eyes were injected with NC as the control group. The 2-mm wide wounds were created in both right and left eyes approximately 6 h after intrastromal siRNA injection as described above. The cornea’s wounded area was demarcated with fluorescein sodium-staining and quantified at 0 h and 24 h with the ImageJ software.

### Chromatin immunoprecipitation (ChIP) assay

HCECs were plated in 6-cm diameter dishes and transfected with either 50 nM SUV39H1 siRNA or NC at approximately 30% confluence. HCECs were harvested 48 h later after siRNA transfection to perform ChIP assay using Magna ChIP™ A/G kit (Merck Millipore) according to the manufacturer’s guidelines. In brief, we sonicated the cross-linked chromatin into 200 to 1000 base pair fragments with Covaris M220 Focused-ultrasonicator™ (Thermo Fisher Scientific). Sheared chromatins were immunoprecipitated using H3K9me3 antibody (Diagenode Inc., pAb-056-050) or SUV39H1 antibody (Merck Millipore, 05615). ChIP-derived DNA was quantified by quantitative PCR using Power SYBR Green PCR Master Mix. With the similar procedure, the corneal epithelium was collected for ChIP assay to evaluate the distributions of H3K9me3 marks at candidate gene promoters during CEWH. Data are analyzed and expressed as a percentage of input signal using the 2^−ΔCt^ method (see Additional file 12: Table S3 for primer sequences).

### Statistical analysis

All data were analyzed using a Student's t-test or one-way ANOVA with Bonferroni correction in multiple groups to determine statistically significant differences. A *P* value less than 0.05 was taken as statistical significance (*P < 0.05, **P < 0.01, ***P < 0.001). All data is provided as mean ± standard error of the mean (SEM).

## Results

### Systematic analysis of epigenetic regulator profile during CEWH

**Fig. 1:**
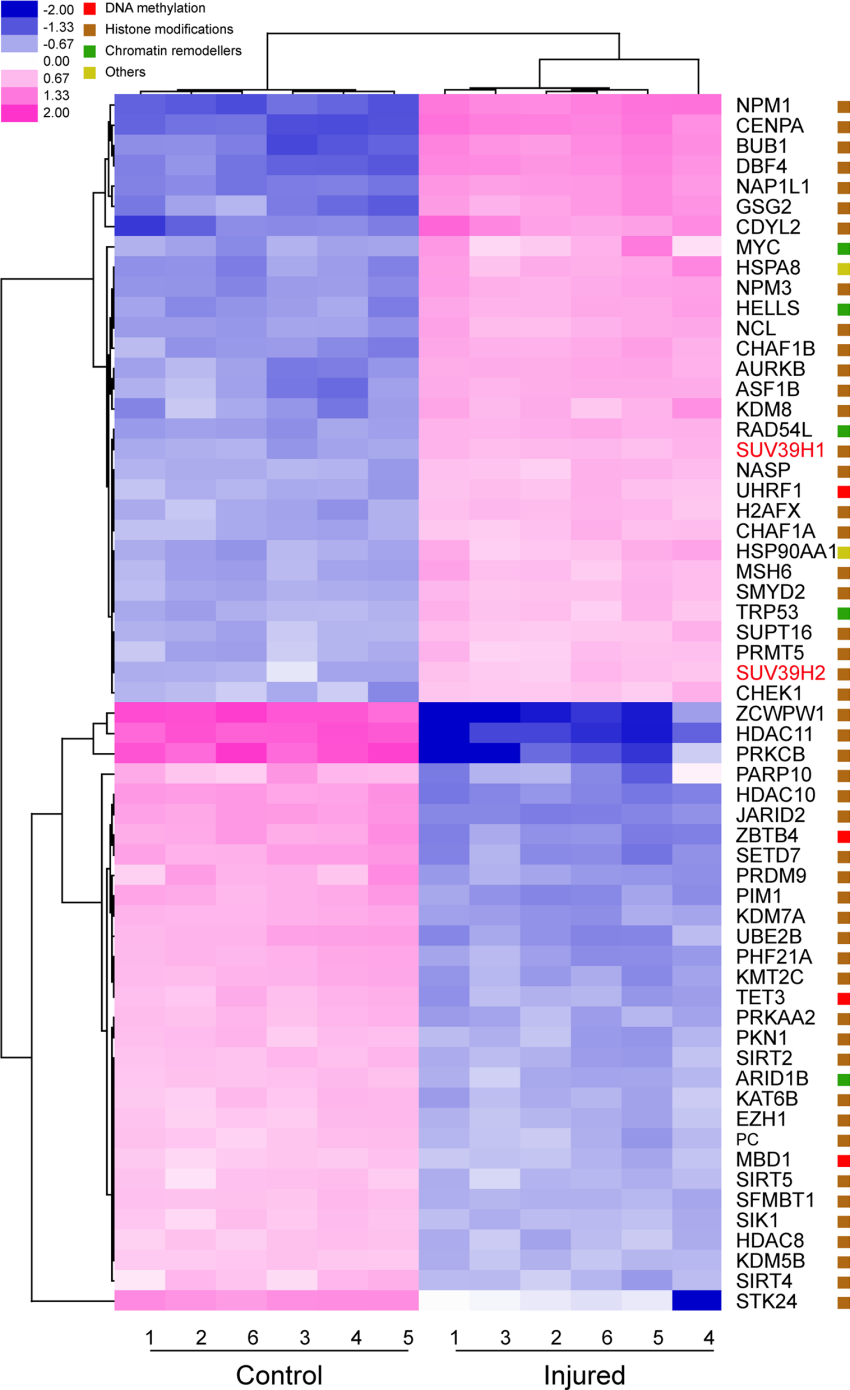
Hierarchical clustering analysis of the 60 most differentially expressed epigenetic modifiers during corneal epithelial wound healing (CEWH). Corneas were subjected to epithelial debridement whereas the control corneas were left untreated. NanoString nCounter technology analyzed the corneal epithelial epigenetic modifier expression profile during CEWH from six mice

Until now, the precise role of epigenetic regulations in CEWH is still incompletely understood. Therefore, we performed a comprehensive analysis to gain insight into the epigenetic regulatory mechanisms underlying this process. To ascertain if epigenetic modifiers are involved in CEWH, we applied NanoString nCounter technology to determine if 336 documented epigenetic modifiers contribute to regulating this process in six mice. We found that 45 epigenetic modifiers were markedly upregulated whereas 47 other epigenetic modifiers were significantly downregulated (Additional file 13: Table S4). The 60 most differentially altered epigenetic modifiers were hierarchically clustered and displayed as a heat map (Fig. 1). As indicated, most of these distinct modifiers are histone modification regulators or chromatin remodeling factors, suggesting that histone modifications and chromatin remodeling play essential roles during CEWH.

### Bioinformatic analyses reveal the potential role of epigenetic regulators in CEWH

We then conducted GO and KEGG pathway enrichment analyses using these differentially expressed epigenetic modifiers. As shown in Additional file 1: Fig. S1, GO enrichment analysis showed that histone modifications were predominant, such as histone H3-K36 methylation, histone arginine methylation, histone H3-K36 demethylation, and histone H3-K9 trimethylation in GO biological process terms. Consistent with these, H3 histone acetyltransferase complex, histone methyltransferase complex, and histone deacetylase complex in GO cellular component terms were enriched during CEWH. Besides, histone demethylase activity (H3-K4 or H3-K36 specific) and histone methyltransferase activity (H3-K9 or H3-K36 specific) in GO molecular function terms were also enriched during CEWH. Furthermore, KEGG pathway enrichment analysis showed that epigenetic modifiers may regulate CEWH through controlling a complex network of various signaling pathways, including those involving cell cycle control, mitogen-activated protein kinase (MAPK), and Wnt signaling pathways (Additional file 2: Fig. S2).

### Both SUV39H1 and SUV39H1 are significantly up-regulated during CEWH

**Fig. 2:**
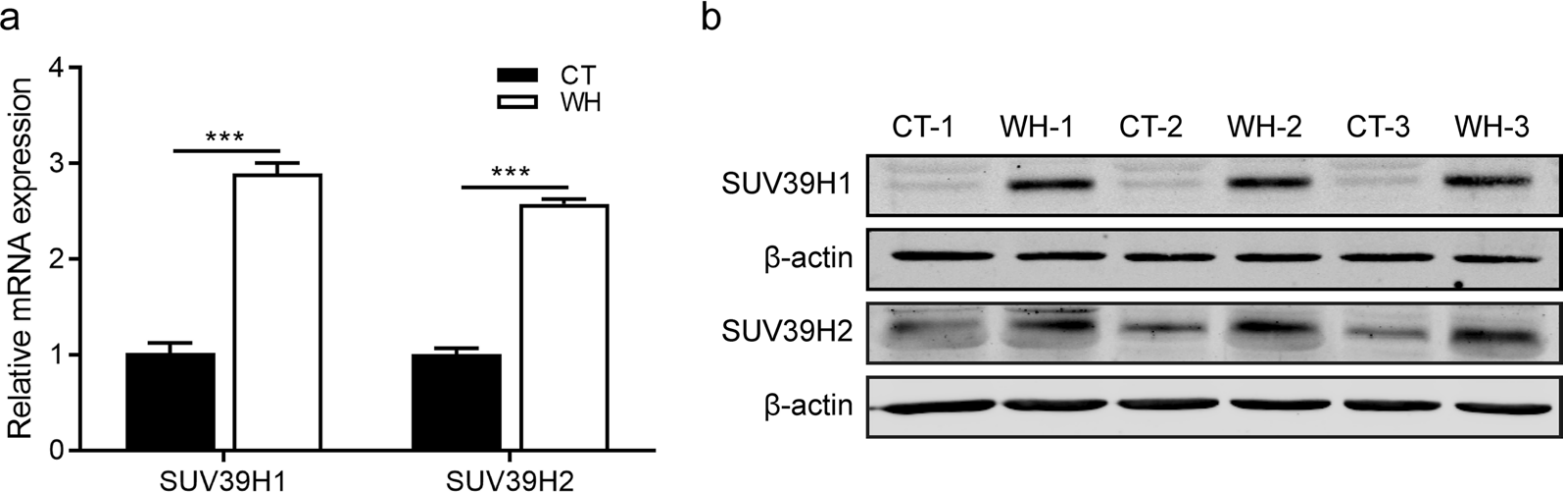
SUV39H1 and SUV39H2 undergo marked upregulation during CEWH. **a** RT-qPCR validated SUV39H1/2 upregulation in wound healing (WH) group compared to that in control (CT) group at 48 h after epithelial debridement (n = 6/group). **b** Western blotting detected the SUV39H1/2 expression levels in the corneal epithelium of WH and CT groups at 48 h after epithelial debridement (n = 3/group)

Among these differentially expressed epigenetic modifiers appearing during CEWH, the significantly upregulated SUV39H1 and SUV39H2 attracted our attention because of their crucial roles in regulating diverse cellular processes via H3K9me3 [18, 21]. RT-qPCR confirmed the upregulation of both SUV39H1 and SUV39H2 mRNA levels during CEWH (Fig. 2a). Such mRNA increases were accompanied by corresponding increases in their protein levels during this process (Fig. 2b).

### SUV39H1 is an effective modulator of HCEC proliferation

**Fig. 3:**
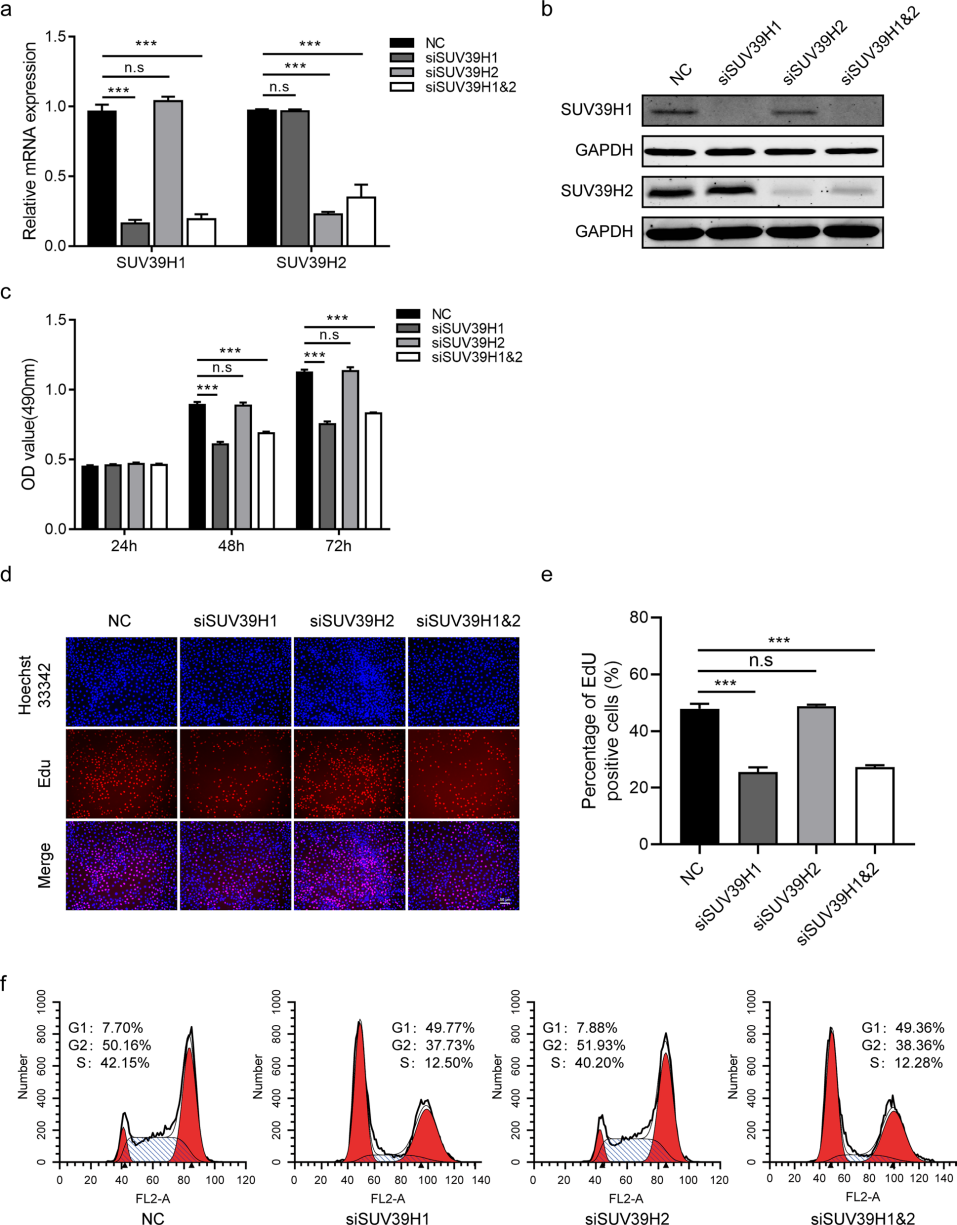
Knockdown of SUV39H1 suppresses human corneal epithelial cell (HCEC) proliferation and induces G1 cell cycle arrest. **a** RT-qPCR measured the mRNA levels of SUV39H1/2 in HCECs at 48 h after transfection with irrelevant negative control (NC) or SUV39H1 and/or SUV39H2 siRNA (n = 3/group). **b** Western blotting examined the protein levels of SUV39H1/2 in HCECs at 48 h after NC or SUV39H1 and/or SUV39H2 siRNA transfection (n = 3/group). **c** MTS assay assessed cell proliferation at different time points after transfection of NC or SUV39H1 and/or SUV39H2 siRNA (n = 6/group). **d** Representative images of EdU staining in HCECs at 48 h after transfection with NC or SUV39H1 and/or SUV39H2 siRNA. Red: EdU; blue: Hoechst 33342; scale bar is 50 μm. **e** Histogram of proliferating HCECs provides values expressed as a percentage of EdU positive cells in NC or SUV39H1 and/or SUV39H2 siRNA transfected HCECs (n = 3/group). **f** Flow cytometry from three independent replicates determined the cell cycle distribution of NC or SUV39H1 and/or SUV39H2 siRNA transfected HCECs at 24 h after release from the second thymidine block. Representative results are shown

To determine the functional roles of SUV39H1/2 during CEWH, we selectively knocked them down by siRNA transfection in HCECs. This procedure reduced SUV39H1 and SUV39H2 mRNA levels by 84% and 77%, respectively, relative to their control levels (Fig. 3a). In agreement with these declines, SUV39H1 and SUV39H2 protein levels were also significantly decreased (Fig. 3b). MTS assay showed that SUV39H1 siRNA transfection significantly reduced HCEC proliferation at 48 h and 72 h of culture, compared with the control group (Fig. 3c). Such declines were confirmed by showing that SUV39H1 knockdown remarkably decreased EdU-positive HCECs (Fig. 3d–e). Furthermore, double thymidine block and release experiments were carried out to evaluate the effect of SUV39H1 knockdown on HCEC cell cycle distribution (Additional file 3: Fig. S3). The decreases in cell proliferation agree with the rise in the proportion of cells arrested at the G1 phase of SUV39H1 siRNA transfected HCECs compared with their proportional distribution pattern in their NC counterpart at 24 h after release from double thymidine block (Fig. 3f). However, SUV39H2 siRNA transfection did not show any significant effects on cell proliferation and cell cycle (Fig. 3c–f). In addition, knockdown of SUV39H1 with another SUV39H1 siRNA also inhibited HCEC proliferation and induced G1 cell cycle arrest (Additional file 4: Fig. S4).

### HCEC migration is unaltered by SUV39H1 or SUV39H2 knockdown

**Fig. 4:**
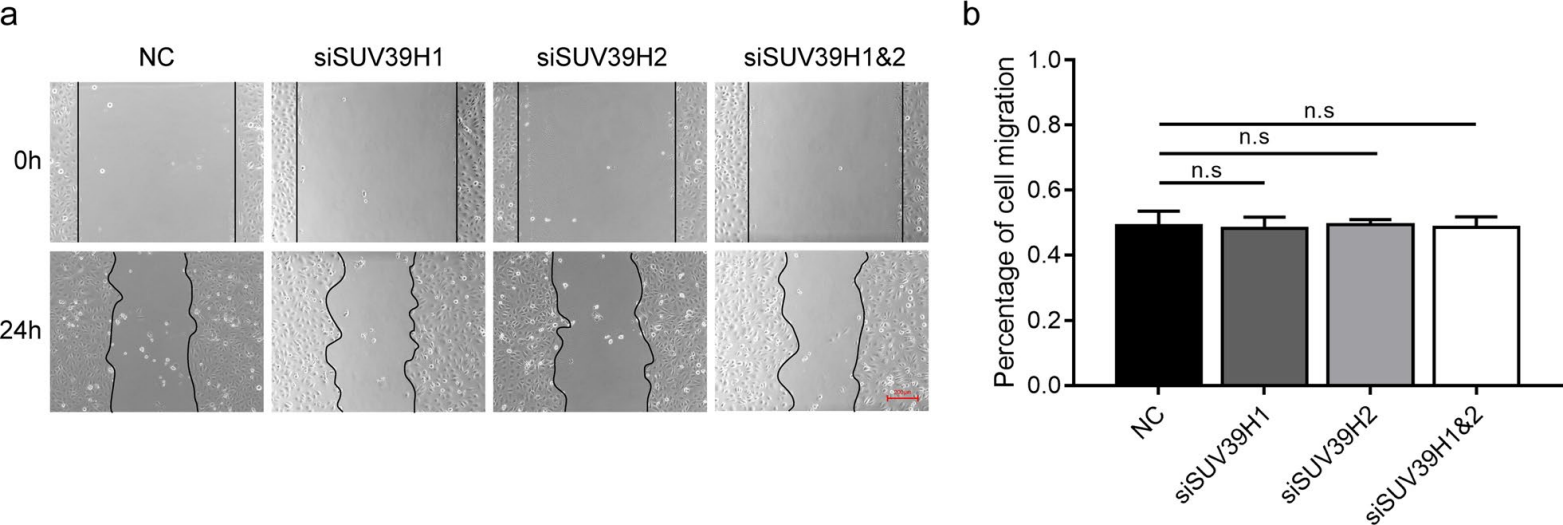
Human corneal epithelial cell (HCEC) migration is unaffected by SUV39H1 and/or SUV39H2 knockdown. **a** The effects of SUV39H1/2 knockdown on cell migration were analyzed by scratch wound assay (n = 3/group). Forty-eight hours after transfection with NC and SUV39H1 and/or SUV39H2 siRNA, HCECs were scratched and cultured for another 24 h in medium without serum. Scale bar is 200 μm. **b** Histogram of HCEC wound closure percentage in NC and SUV39H1 and/or SUV39H2 siRNA transfected HCECs (n = 5/group)

To evaluate the effects of SUV39H1 and SUV39H2 on HCEC migration, we determined whether the time required for wound closure is different between SUV39H1 or SUV39H2 siRNA transfected cells and the NC group. The results showed no significant difference in cell migration between either the SUV39H1 knockdown or the SUV39H2 knockdown groups and their corresponding NC group (Fig. 4).

### Suppression of SUV39H1 delays in vivo CEWH

**Fig. 5:**
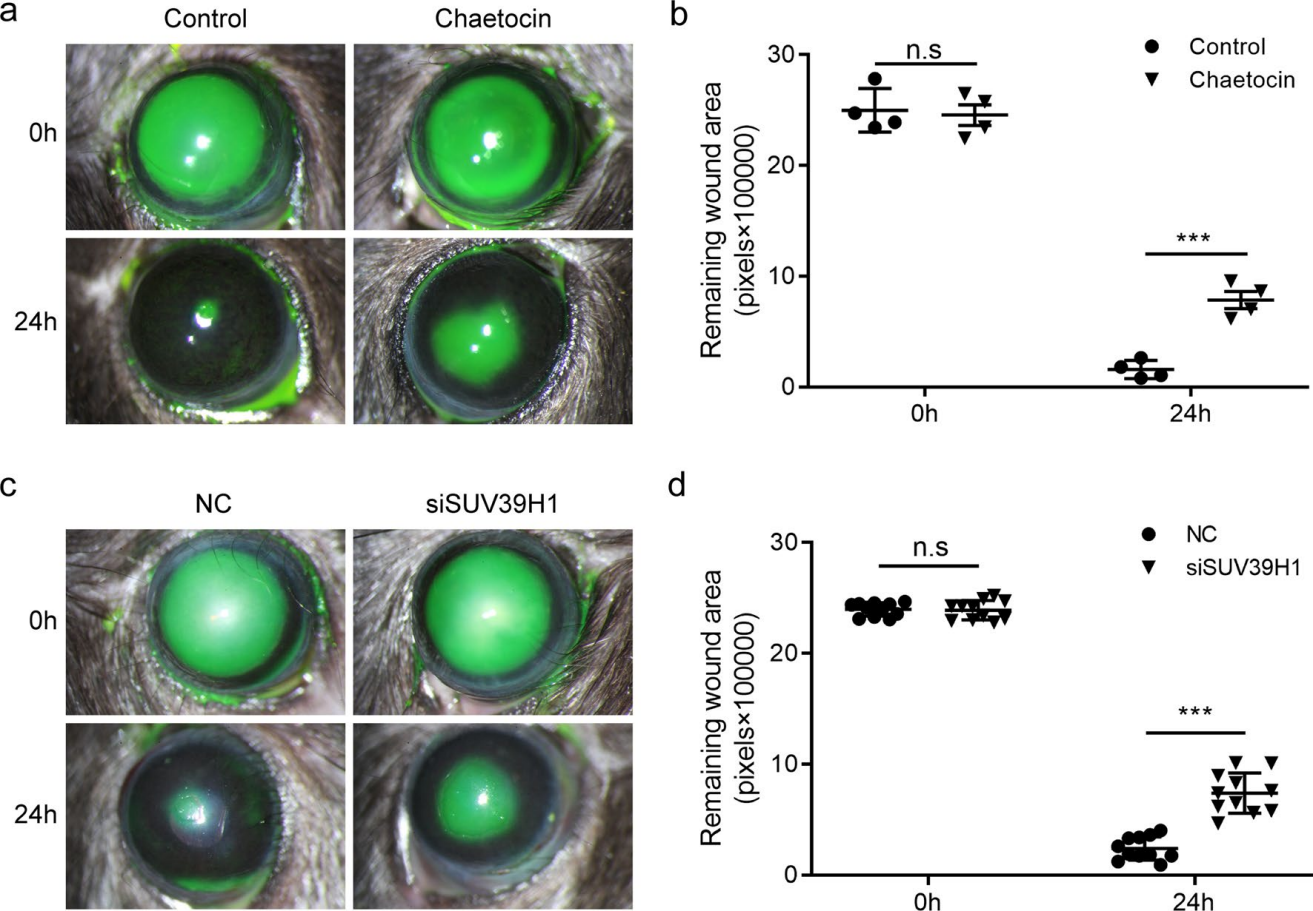
Loss-of-function of SUV39H1 remarkably retards corneal epithelial wound healing (CEWH) in mice. **a** Chaetocin dramatically delayed corneal epithelial wound closure. Representative images of fluorescein sodium-stained corneas in chaetocin and physiological saline treated groups are shown. **b** Histogram of residual epithelial defects of corneas in the chaetocin or physiological saline treated groups (n = 4/group). The remaining unrepaired areas are presented in pixels of size. **c** Repression of SUV39H1 expression markedly delayed corneal epithelial wound closure. Representative images are shown of fluorescein sodium-stained corneas in SUV39H1 siRNA or NC injected groups. **d** Histograms of residual epithelial defects of corneas in SUV39H1 siRNA and NC injected groups (n = 11/group). The remaining wound areas are presented in pixels of size

To explore the physiological relevance of the results showing that SUV39H1 is an epigenetic modulator of CEWH in vitro, we determined if an inhibitor of SUV39H1 enzymatic activity, chaetocin, alters CEWH in vivo. A corneal epithelial debridement wound was created in both right and left murine eyes, respectively. Then, chaetocin was instilled into the right eyes every 2 h whereas the contralateral control eyes were treated with physiological saline. Strikingly, the wound closure was delayed in the chaetocin-treated corneas compared to the control group (Fig. 5a, b). Furthermore, we confirmed this delay in wound closure by showing that the remaining wound area was larger in the SUV39H1 siRNA-injected corneas than the NC (Fig. 5c, d). The efficiency of siRNA-mediated knockdown was confirmed by RT-qPCR analysis (Additional file 5: Fig. S5). Similarly, injection with a different siRNA against SUV39H1 into the cornea also reduced SUV39H1 expression and remarkedly delayed corneal epithelial wound closure (Additional file 6: Fig. S6). Taken together, these data demonstrated that SUV39H1 loss of function effectively inhibits CEWH in vivo.

### SUV39H1 modulates cell cycle progression through targeting p27 and p21

**Fig. 6:**
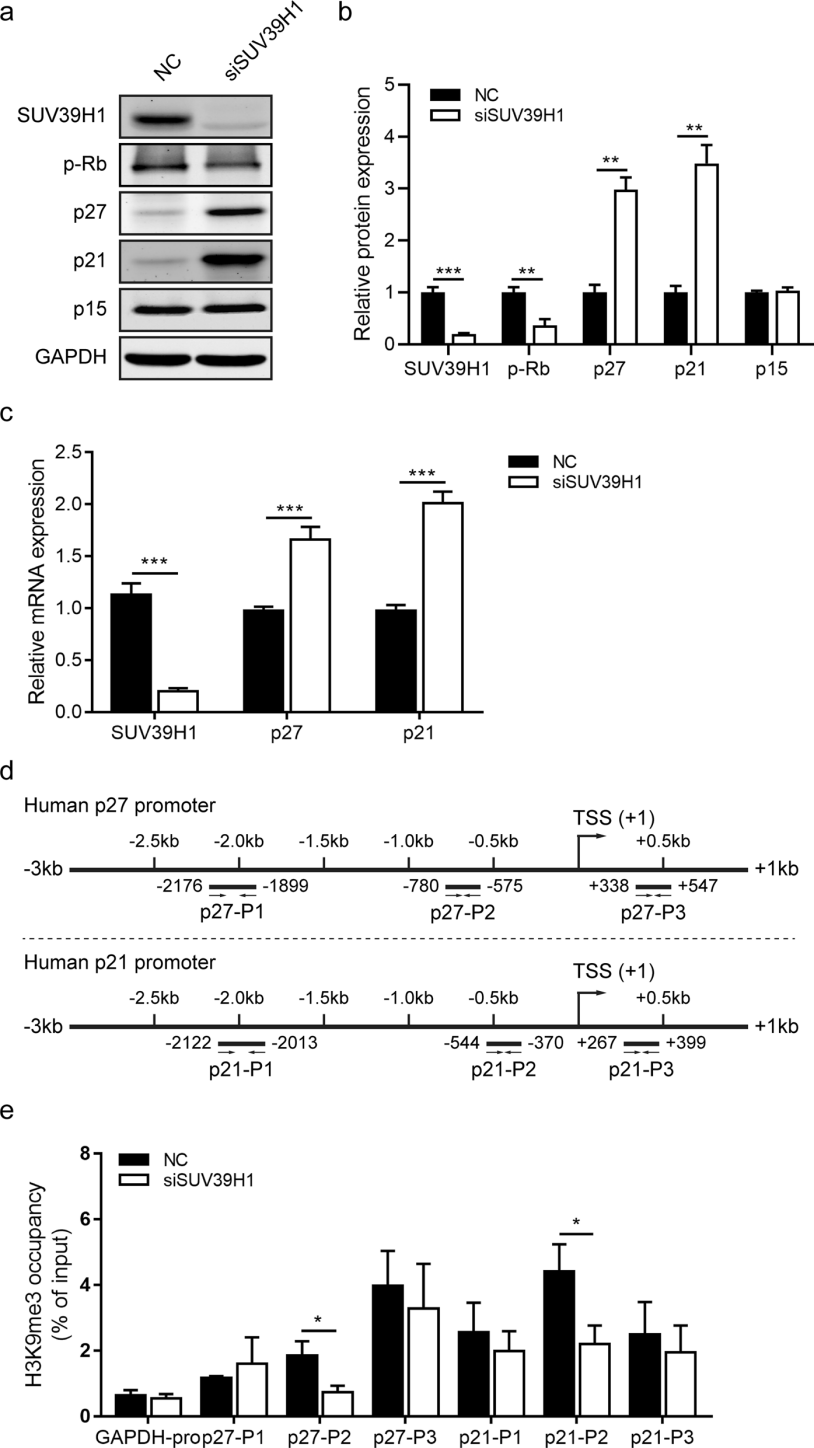
SUV39H1 knockdown upregulates p27 and p21 expression via reducing H3K9me3 marks at their promoters. **a** Western blotting was performed to measure protein levels of cell cycle regulators at 48 h after siRNA transfection in human corneal epithelial cells (HCECs) (n = 3/group). **b** Histogram of the quantified protein expression of cell cycle regulators in HCECs after siRNA transfection (n = 3/group). GAPDH is a normalized control. **c** RT-qPCR was used to assess SUV39H1, p27, and p21 expression levels at 48 h after siRNA transfection in HCECs (n = 3/group). **d** Diagrammatic representation provides the design of primers spanning different regions of human p27 and p21 promoters. **e** Chromatin immunoprecipitation-quantitative polymerase chain reaction (ChIP-qPCR) was used to analyze the H3K9me3 marks at the p27 and p21 promoters at 48 h after siRNA transfection in HCECs (n = 3/group). Results are expressed as percentage (%) of input signal

To clarify how SUV39H1 knockdown inhibits cell cycle progression and proliferation of HCECs, we determined its effect on the expression of key proteins associated with regulating G1-S transition [34]. p27 and p21 were identified as candidate genes for further analysis (Additional file 7: Fig. S7). As shown in Fig. 6a, b, p27, and p21 protein levels were upregulated in SUV39H1 siRNA-transfected HCECs compared with the NC. Moreover, knockdown of SUV39H1 downregulated p-Rb, but the levels of p15 were nearly invariant. In addition, SUV39H1 repression also reduced cyclin D1 and CDK6 in HCECs (Additional file 8: Fig. S8). Furthermore, downregulation of p-Rb and CDK6 in the SUV39H1 siRNA-transfected HCECs was confirmed by FACS (Additional file 8: Fig. S8). Given that SUV39H1 suppresses gene transcription via H3K9me3 [23, 24], we assumed that p27 and p21 are candidate target genes of SUV39H1 in HCECs. As predicted, SUV39H1 siRNA transfection significantly increased p21 and p27 mRNA levels when compared with NC (Fig. 6c). Furthermore, ChIP assay demonstrated that SUV39H1 knockdown significantly reduced H3K9me3 marks at the p27 promoter (− 780 to − 575 nt) and p21 promoter (− 544 to − 370 nt) around the transcription start sites (Fig. 6d, e), consistent with the reduced SUV39H1 binding at their promoters (Additional file 9: Fig. S9).

### SUV39H1 represses p27 via increasing H3k9me3 marks at its promoter during CEWH

**Fig. 7:**
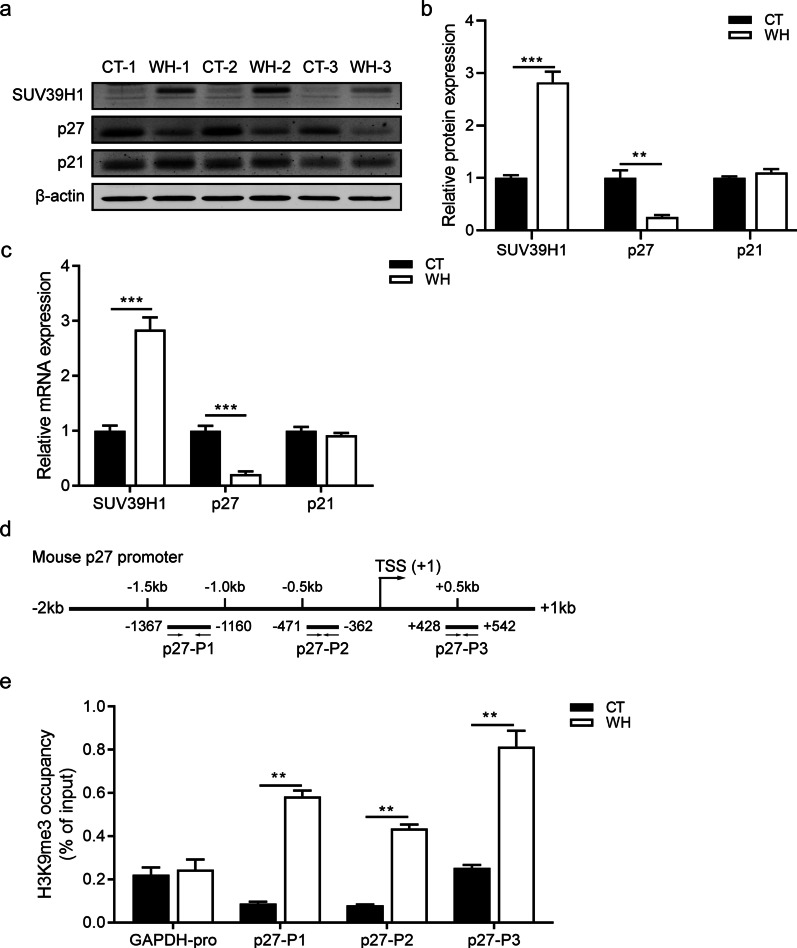
SUV39H1 represses p27 expression level during corneal epithelial wound healing (CEWH) in mice. **a** Western blotting analysis shows SUV39H1, p27, and p21 protein levels in corneal epithelium in wound healing (WH) and control (CT) groups at 48 h after injury (n = 3/group). **b** Densitometric quantification of SUV39H1, p27, and p21 protein expression in CEWH was performed (n = 3/group). **c** RT-qPCR analysis shows SUV39H1, p27, and p21 mRNA levels in corneal epithelium of WH and CT groups at 48 h after injury (n = 4/group). **d** Graphical illustration of the design of primers spanning different regions of the mouse p27 promoter. **e** Chromatin immunoprecipitation-quantitative polymerase chain reaction (ChIP-qPCR) was used to analyze the H3K9me3 marks at the p27 promoter site in the corneal epithelium in WH and CT groups at 48 h after injury from three independent replicates. Results are presented as percentage (%) of input signal

To confirm whether SUV39H1 regulates p27 and p21 during CEWH, we performed Western blotting and RT-qPCR analysis. The results indicated that the p27 expression level significantly declined in the wound healing group, whereas SUV39H1 mRNA and protein levels were upregulated when compared with the control group (Fig. 7a–c). On the other hand, the p21 expression level appeared unaltered. Furthermore, the H3K9me3 marks at different regions of the p27 promoter in the corneal epithelium were significantly upregulated after injury compared with the normal control (Fig. 7d–e). Overall, our data establish a regulatory mechanism of SUV39H1-mediated repression of p27 during CEWH both in vitro and in vivo.

## Discussion

Corneal epithelial renewal is a complex response entailing time-dependent expression modulation of numerous genes controlling cell proliferation, migration, and differentiation [4, 35]. Emerging evidence suggests that different epigenetic mechanisms contribute to the control of gene expression [36, 37], which are precisely implemented by distinct epigenetic modifiers. Recent studies have revealed that DNA methylation and miRNAs indeed regulate CEWH [10, 13, 38]. However, there are few reports systematically describing the roles of histone modifier alteration in modulating this process.

In a previous study, we adopted NanoString nCounter technology to characterize an accurate miRNA signature during CEWH. Here, we expanded our interest to include a more comprehensive portfolio of epigenetic modifiers. The NanoString nCounter analysis precisely identified 92 epigenetic regulators that were significantly altered during CEWH. Among these epigenetic modifiers, UHRF1 and TET3 are associated with DNA methylation [39, 40], suggesting a dynamic change of DNA methylation is also involved in CEWH. In addition, histone H3 variant CENPA and histone H2 variant H2AFX underwent upregulation during CEWH. Besides, corneal injury upregulated KDM8 which functions as a transcriptional activator by demethylating H3K36me2 [41]. Interestingly, KDM8 was also overexpressed during skin injury repair in rats [42]. In addition, histone deacetylases (HDACs) such as HDAC11 and HDAC10 are markedly downregulated in response to corneal injury, suggesting that histone acetylation mediated gene activation is important for CEWH. In tandem with this, HDAC inhibitor valproic acid can promote spinal cord injury repair by upregulating the expressions of Hsp70 and Bcl-2 in rats [43]. Similarly, another HDAC inhibitor, trichostatin A can accelerate the digit regeneration in mice [44]. Specifically, corneal injury resulted in an upregulation of histone methyltransferase SUV39H1 and SUV39H2, which control the methylation status of H3K9me3 [45], a well-established epigenetic repressive mark. To the best of our knowledge, this study is the first to provide a comprehensive and precise epigenetic modifier signature during CEWH. Such data will assuredly be a valuable resource for researchers in this field.

Further GO analysis demonstrated that most of these epigenetic modifiers were correlated with histone covalent modifications, such as histone H3-K9 trimethylation, suggesting that histone modifications are crucial contributors in regulating CEWH. Additionally, KEGG pathway analysis showed that the WNT and MAPK signaling pathways and cell cycle progression were activated in response to corneal epithelial injury, which is consistent with our previous report [13]. Therefore, our data demonstrated that corneal injury stimulates the alteration of epigenetic modifier expression, which suggests that epigenetic mechanisms are widely involved in regulating CEWH.

Previous studies demonstrated that SUV39H1/2 can regulate cell proliferation and migration [25, 46–48]. Both of these responses are vital for the rate of CEWH process [4]. One of our approaches to characterize the role of SUV39H1/2 in mediating CEWH entailed determining the effects of the loss of SUV39H1/2 function on HCEC proliferation and migration. MTS and EdU staining demonstrated that SUV39H1 silencing significantly inhibited HCEC proliferation. These declines are consistent with the retardation of cell cycle progression because downregulation of SUV39H1 increased the proportion of HCECs in the G1 phase. These effects agree with those showing that SUV39H1 knockdown also inhibited proliferation in other cell lines, such as GOS-3 and T98G glioma cells [49]. It is noteworthy that SUV39H2 knockdown had no inhibitory effects on either HCEC proliferation or cell cycle progression, which may be caused by the diverse tissue-specific effects of SUV39H1/2 in different cells and tissues. Yokoyama et al. found that overexpression of SUV39H1 activated migration in breast and colorectal cancer cells [50]. This finding is partly consistent with another recent report in which knockdown of Suv39H1 restored E-cadherin expression and inhibited cell migration of basal-like breast cancer [25]. In addition, Huang et al. revealed that SUV39H2 contributes to cell proliferation and metastasis of colon cancer via increasing H3K9me3 marks at the SLIT1 promoter [48]. Above all, we identified that SUV39H1 acts as an epigenetic regulator of HCEC proliferation rather than SUV39H2. Moreover, our results suggest that wound-induced SUV39H1 upregulation mediates increases in mouse corneal epithelial cell proliferation in vivo.

In 2005, Greiner et al. showed that chaetocin selectively inhibited histone methyltransferase SU(VAR)3–9 over a narrow concentration range [51]. In this study, we found that chaetocin can significantly delay mouse CEWH, which supports the notion that SUV39H1 has an essential role in stimulating corneal epithelial proliferation and wound healing. However, chaetocin was also reported to inhibit another histone methyltransferase G9a besides SUV39H1 at higher concentrations which may blunt increases in corneal epithelial cell proliferation [52, 53]. To confirm the involvement of SUV39H1 in modulating cell proliferation and CEWH, these responses to injury were further evaluated in vivo using SUV39H1 siRNA injection to decrease its expression. This procedure dramatically retarded corneal wound closure where we saw a 50% decline in the expression of SUV39H1 after specific siRNA injection, supporting the notion that elevated SUV39H1 expression has a direct effect on promoting CEWH in mice.

It is well-known that cell proliferation is dependent on cell cycle control [34, 54]. Chung et al. found that corneal epithelial debridement stimulates synchronization of the G1/S transition of basal cells into the cell cycle [55]. This finding is partly consistent with our result that silencing SUV39H1 led to G1 cell cycle arrest in HCECs. Progression through the G1 phase and G1/S checkpoint is dependent on CDK4, CDK6, and CDK2 activation, as well as their interactions with corresponding cyclin co-factors. On the other hand, CDKIs such as p27 and p21 can effectively inhibit cyclin-CDK complex activity, and thus impede transit through the G1/S checkpoint [34]. In this study, SUV39H1 knockdown led to the upregulation of p27 and p21, and downregulation of Cyclin D1 and CDK6 in HCECs. These alterations then suppressed transit through the G1/S checkpoint through a decrease in Rb phosphorylation in HCECs, which is a critical mediator in the control of cell proliferation [56, 57]. It is well-established that the repressive control of gene expression by SUV39H1 is mediated through modulating H3K9me3 marks on the target gene promoters [22–24]. In a previous study, SUV39H1 was recruited to the p21 gene promoter and repressed its expression via increasing H3K9me3 marks in microglial cells [27]. Consistent with these findings, we observed that knockdown of SUV39H1 significantly reduced H3K9me3 marks and SUV39H1 binding at the p27 and p21 promoters around transcription start sites in HCECs. The importance of p27 modulation to the control of proliferation during CEWH is substantiated by another report showing that p15 and p27 expression levels dramatically declined while p21 expression did not appear to change after corneal injury [58]. Indeed, the downregulation of p27 expression accompanied SUV39H1 upregulation during CEWH. In parallel with this reciprocal relationship between changes in SUV39H1 and p27 expression levels, the H3K9me3 marks at p27 promoter were significantly increased during CEWH, suggesting that p27 is indeed a target gene of SUV39H1. To sum up, our findings reveal that wound-induced increase in SUV39H1 expression has a corresponding effect on cell proliferation through H3K9me3-mediated repression of p27 both in vitro and in vivo. Further studies are warranted to elucidate the detailed mechanism that accounts for how SUV39H1 affects p27 expression through modulating H3K9me3 marks at the p27 promoter during CEWH.

## Conclusions

We identify a comprehensive signature of epigenetic modifiers during CEWH in mice. Upregulation of SUV39H1 promotes CEWH through regulating cell proliferation which is mediated through increased H3K9me3 marks at the p27 promoter and in turn suppressed its expression. Our findings point to the possibility that epigenetic modifiers such as SUV39H1 can be served as potential targets for promoting CEWH.

## Supplementary Information

**Additional file 1: Fig. S1.** Gene ontology (GO) enrichment analysis of differentially expressed epigenetic modifiers during corneal epithelial wound healing (CEWH). The top 15 most significant enriched GO functional terms are shown. The X-axis shows different gene function terms, including biological process (red), cellular component (blue), and molecular function (green).

**Additional file 2: Fig. S2.** Kyoto Encyclopedia of Genes and Genomes (KEGG) pathway analysis of altered epigenetic modifiers during corneal epithelial wound healing (CEWH). The top 10 most enriched signaling pathways in differentially expressed epigenetic modifiers are shown. The Y-axis shows different signaling pathways. Bubbles of various sizes and hues represent the corresponding amounts of altered epigenetic modifiers enriched in a signaling pathway and their significance.

**Additional file 3: Fig. S3.** SUV39H1 knockdown leads to G1 phase arrest in human corneal epithelial cells (HCECs). HCECs transfected with NC or SUV39H1 siRNA were cultured in DMEM/F12 medium containing 2 mmol/L thymidine for 16 h. Then, HCECs were cultured in normal DMEM/F12 medium without thymidine for 9 h after twice PBS washing. After an additional twice PBS wash, HCECs were again subjected to 2 mmol/L thymidine for 16 h. After twice PBS wash, HCECs were again cultured in normal medium to be collected at the relevant time points after being released from the thymidine block to detect cell cycle distribution via flow cytometry. Representative results are shown.

**Additional file 4: Fig. S4.** Knockdown of SUV39H1 via another siRNA inhibits human corneal epithelial cell (HCEC) proliferation and induces G1 cell cycle arrest. **a** The mRNA levels of SUV39H1, p27, and p21 in HCECs were measured at 48 h after transfection with irrelevant negative control (NC) or SUV39H1 siRNA (n = 3/group) via RT-qPCR. **b** The protein levels of cell cycle regulators were detected at 48 h after siRNA transfection in HCECs by Western blotting. **c** Densitometric Western blotting analysis quantifying the protein expression in transfected HCECs was performed (n = 3/group). **d** MTS assay evaluated cell proliferation in HCECs with the transfection of NC or SUV39H1 siRNA at different time points (n = 6/group). **e** Flow cytometry determined the effect of SUV39H1 on cell cycle distribution in HCECs at 24 h after release from the second thymidine block. Representative results are shown. **f** Representative images of EdU staining in HCECs transfected with NC or SUV39H1 siRNA. Red: EdU; blue: Hoechst 33342; scale bar is 50 μm. **g** Histogram of the percentage of EdU positive cells showing the proliferating cells in NC or SUV39H1 siRNA transfected HCECs (n = 4/group).

**Additional file 5: Fig. S5.** SUV39H1 expression decreased with the presence of SUV39H1 siRNA in murine corneal epithelium during corneal epithelial wound healing (CEWH). RT-qPCR was used to analyze the expression levels of SUV39H1 in the negative control (NC) and the SUV39H1 siRNA-injected groups (n = 3/group).

**Additional file 6: Fig. S6.** SUV39H1 repression with a different siRNA remarkedly delays corneal epithelial wound closure. **a** The expression level of SUV39H1 in the corneal epitheliums of negative control (NC) and the SUV39H1 siRNA-injected groups were quantified by RT-qPCR (n = 4/group). **b** Representative images of fluorescein sodium-stained corneas in SUV39H1 siRNA or NC injected groups. **c** Scatter plots of residual epithelial defects of corneas in SUV39H1 siRNA and NC injected groups (n = 7/group). The remaining wound areas are presented in pixels of size.

**Additional file 7:Fig. S7.** Expression of cell cycle associated genes in human corneal epithelial cells (HCECs) transfected with SUV39H1 siRNA. RT-qPCR was used to systematically analyze the alteration of cell cycle associated genes in HCECs at 48 h after transfection with negative control (NC) or SUV39H1 siRNA.

**Additional file 8: Fig. S8.** SUV39H1 repression significantly decreases the phosphorylation level of Rb, Cyclin D1, and CDK6 in human corneal epithelial cells (HCECs). **a** Western blotting detected the protein levels of Cyclin D1, CDK2, CDK4, and CDK6 in NC or SUV39H1 siRNA transfected HCECs. **b** The protein levels of Cyclin D1 and CDKs were quantified from three independent replicates. **c** Histogram of normalized mean fluorescence intensity of CDK6 in NC siRNA or SUV39H1 siRNA transfected HCECs by fluorescence activated cell sorting (FACS) (n = 3/group). **d** Representative result of CDK6 in FACS is shown in HCECs after siRNA transfection. **e** Histogram of normalized positive p-Rb cells in negative control (NC) or SUV39H1 siRNA transfected HCECs (n = 4/group) via FACS. The percentage of positive p-Rb cells in HCECs transfected with NC siRNA acted as the normalized control. **f** Representative line chart of the positive p-Rb cells in HCECs transfected with NC or SUV39H1 siRNA via FACS.

**Additional file 9: Fig. S9.** SUV39H1 directly binds to the p27 and p21 promoters in human corneal epithelial cells (HCECs). Chromatin immunoprecipitation-quantitative polymerase chain reaction (ChIP-qPCR) was performed to analyze SUV39H1 occupancy at the p27 and p21 promoters at 48 h after siRNA transfection in HCECs (n = 3/group). The percentage (%) of the input signal is used to express the results.

**Additional file 10: Table S1.** Genes included in NanoString probe set.

**Additional file 11: Table S2.** Primers used for RT-qPCR analysis.

**Additional file 12: Table S3.** ChIP-qPCR primers used for candidate gene promoters.

**Additional file 13: Table S4.** Differentially expressed epigenetic modifiers during CEWH.

## Data Availability

All data generated or analyzed during this study are included in this published article along with the relevant supplementary files.

## Abbreviations

CEWH: Corneal epithelial wound healing
SUV39H1: Suppressor of variegation 3-9 homolog 1
H3K9me3: Histone H3 lysine 9 trimethylation
HCEC: Human corneal epithelial cell
ncRNAs: Non-coding RNAs
SAHA: Suberoylanilide hydroxamic acid
CDKI: Cyclin-dependent kinase inhibitor
FDR: False discovery rate
GO: Gene ontology
KEGG: Kyoto Encyclopedia of Genes and Genomes
RT-qPCR: Quantitative reverse transcription polymerase chain reaction
cDNA: Complementary DNA
NC: Negative control
DTB: Double thymidine block
PI: Propidium iodide
FACS: Fluorescence activated cell sorting
ChIP: Chromatin immunoprecipitation
SEM: Standard error of the mean
MAPK: Mitogen-activated protein kinase
HDACs: Histone deacetylases
