# Supplementary figures and images for: SUV39H1 regulates corneal epithelial wound healing via H3K9me3-mediated repression of p27
Source: Eye Vis (Lond). 2022 Feb 1;9:4. doi: 10.1186/s40662-022-00275-5 (PMC8805298; doi:10.1186/s40662-022-00275-5)

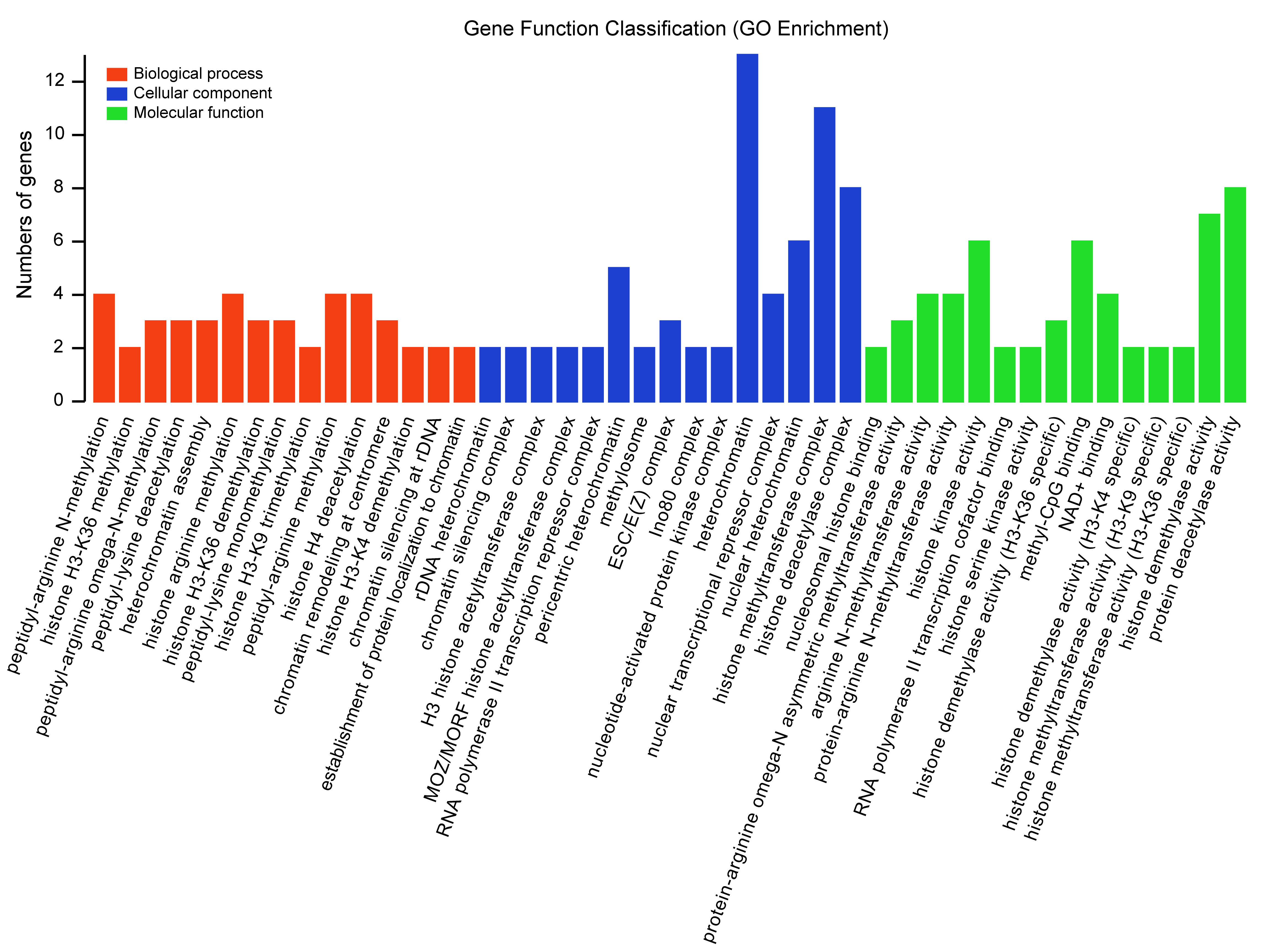

Supplement: Supplementary file 1 — Additional file 1: Fig. S1. Gene ontology (GO) enrichment analysis of differentially expressed epigenetic modifiers during corneal epithelial wound healing (CEWH). The top 15 most significant enriched GO functional terms are shown. The X-axis shows different gene function terms, including biological process (red), cellular component (blue), and molecular function (green). [file 40662_2022_275_MOESM1_ESM.tif]

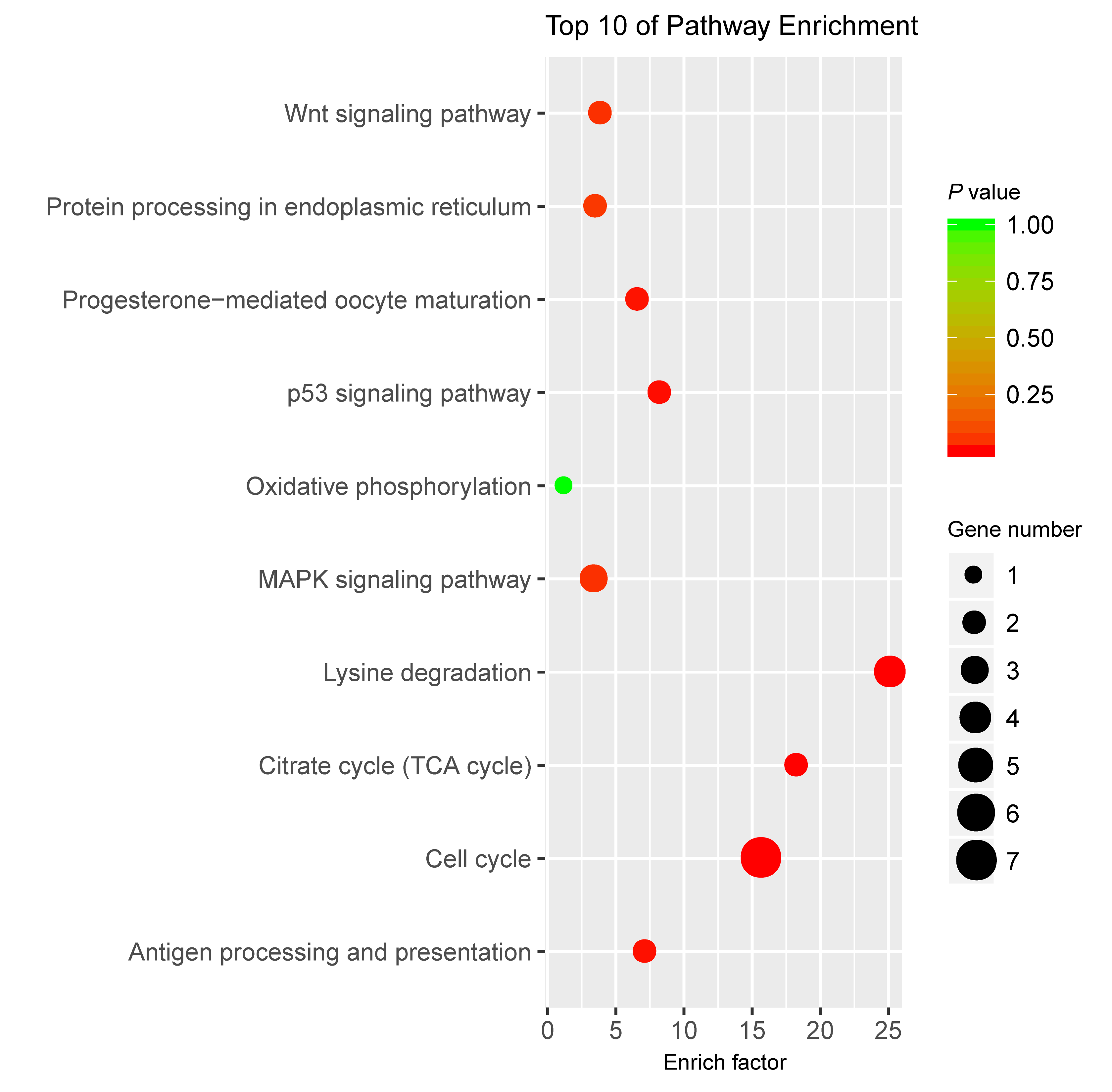

Supplement: Supplementary file 2 — Additional file 2: Fig. S2. Kyoto Encyclopedia of Genes and Genomes (KEGG) pathway analysis of altered epigenetic modifiers during corneal epithelial wound healing (CEWH). The top 10 most enriched signaling pathways in differentially expressed epigenetic modifiers are shown. The Y-axis shows different signaling pathways. Bubbles of various sizes and hues represent the corresponding amounts of altered epigenetic modifiers enriched in a signaling pathway and their significance. [file 40662_2022_275_MOESM2_ESM.tif]

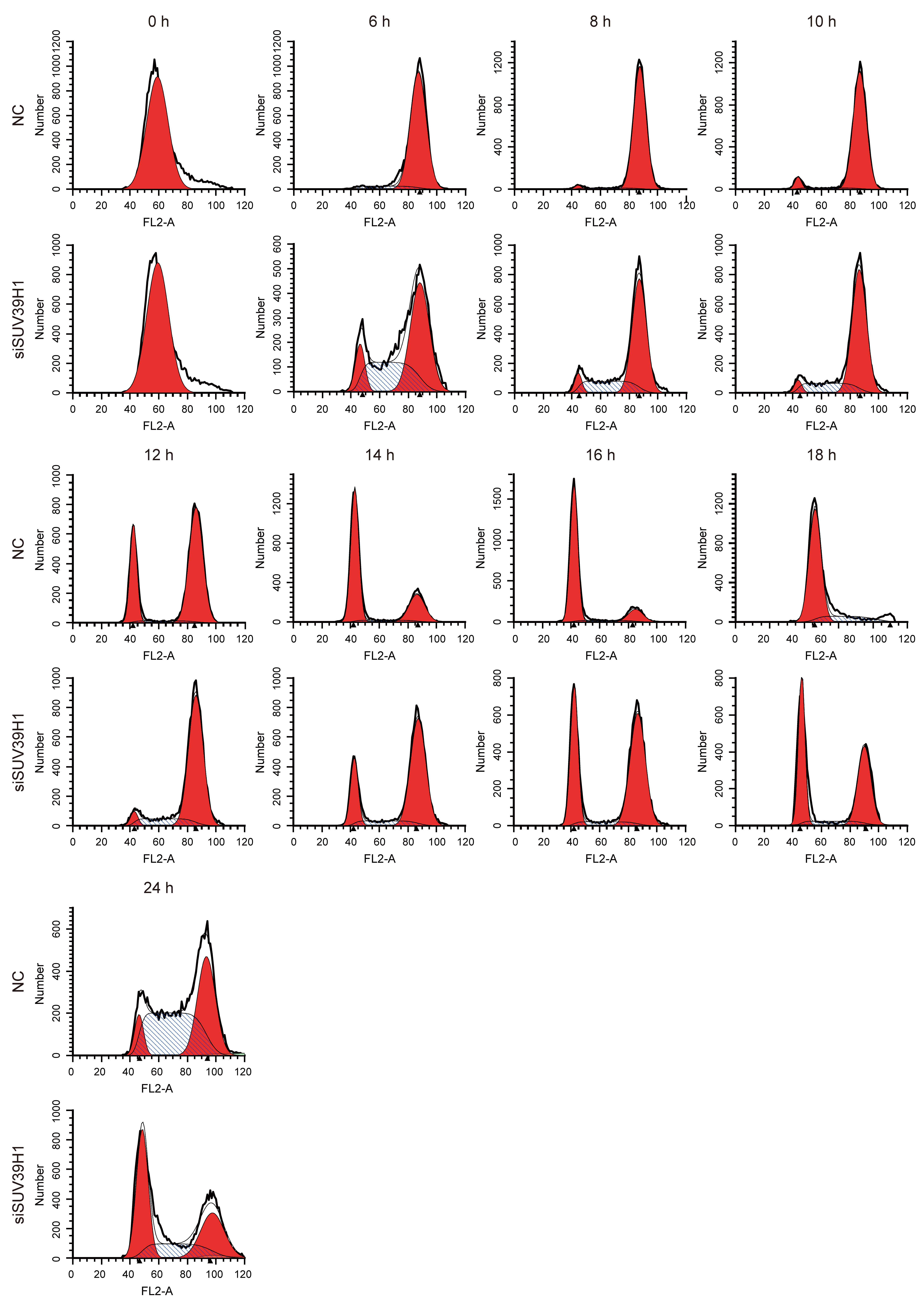

Supplement: Supplementary file 3 — Additional file 3: Fig. S3. SUV39H1 knockdown leads to G1 phase arrest in human corneal epithelial cells (HCECs). HCECs transfected with NC or SUV39H1 siRNA were cultured in DMEM/F12 medium containing 2 mmol/L thymidine for 16 h. Then, HCECs were cultured in normal DMEM/F12 medium without thymidine for 9 h after twice PBS washing. After an additional twice PBS wash, HCECs were again subjected to 2 mmol/L thymidine for 16 h. After twice PBS wash, HCECs were again cultured in normal medium to be collected at the relevant time points after being released from the thymidine block to detect cell cycle distribution via flow cytometry. Representative results are shown. [file 40662_2022_275_MOESM3_ESM.tif]

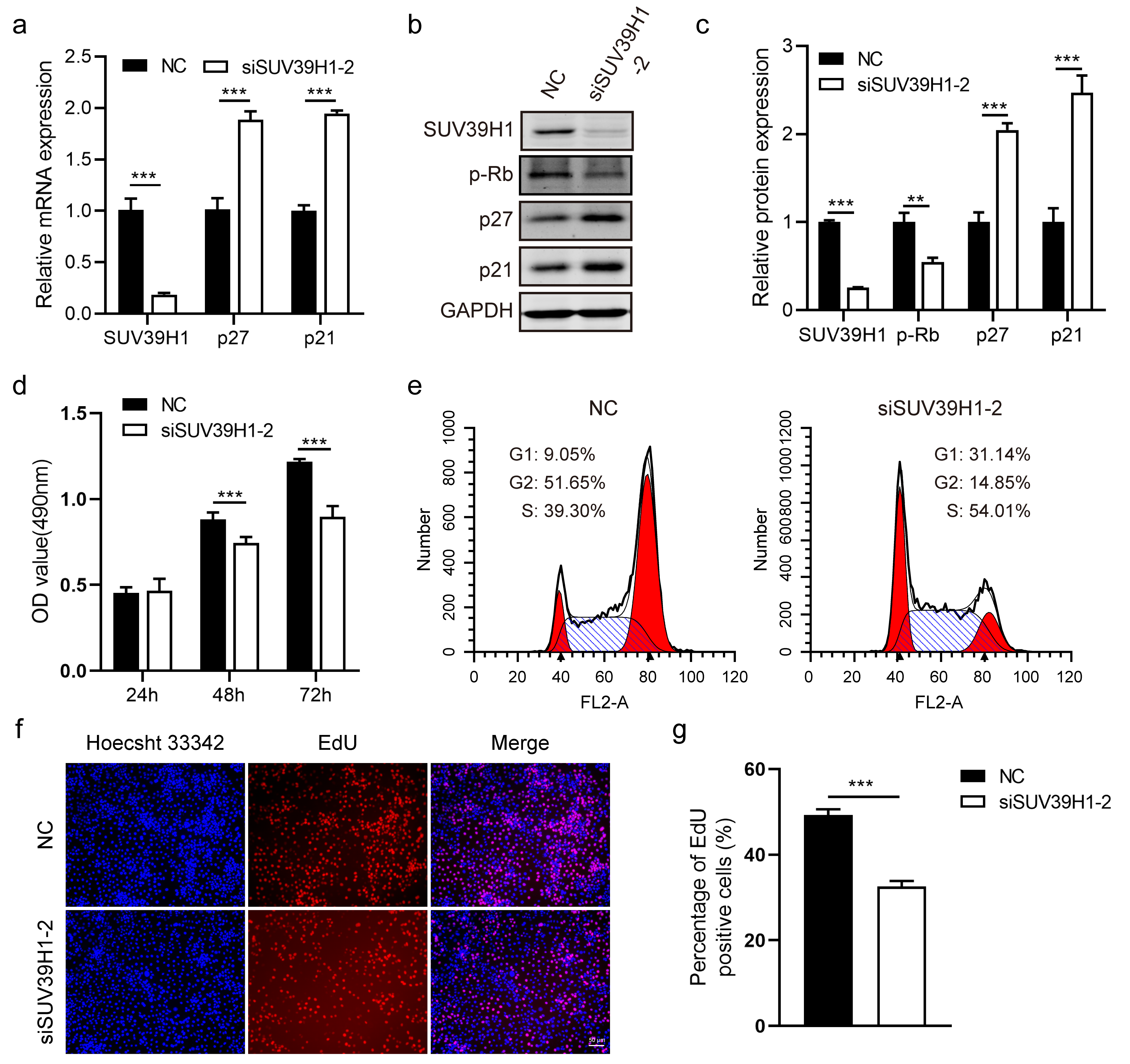

Supplement: Supplementary file 4 — Additional file 4: Fig. S4. Knockdown of SUV39H1 via another siRNA inhibits human corneal epithelial cell (HCEC) proliferation and induces G1 cell cycle arrest. a The mRNA levels of SUV39H1, p27, and p21 in HCECs were measured at 48 h after transfection with irrelevant negative control (NC) or SUV39H1 siRNA (n = 3/group) via RT-qPCR. b The protein levels of cell cycle regulators were detected at 48 h after siRNA transfection in HCECs by Western blotting. c Densitometric Western blotting analysis quantifying the protein expression in transfected HCECs was performed (n = 3/group). d MTS assay evaluated cell proliferation in HCECs with the transfection of NC or SUV39H1 siRNA at different time points (n = 6/group). e Flow cytometry determined the effect of SUV39H1 on cell cycle distribution in HCECs at 24 h after release from the second thymidine block. Representative results are shown. f Representative images of EdU staining in HCECs transfected with NC or SUV39H1 siRNA. Red: EdU; blue: Hoechst 33342; scale bar is 50 μm. g Histogram of the percentage of EdU positive cells showing the proliferating cells in NC or SUV39H1 siRNA transfected HCECs (n = 4/group). [file 40662_2022_275_MOESM4_ESM.tif]

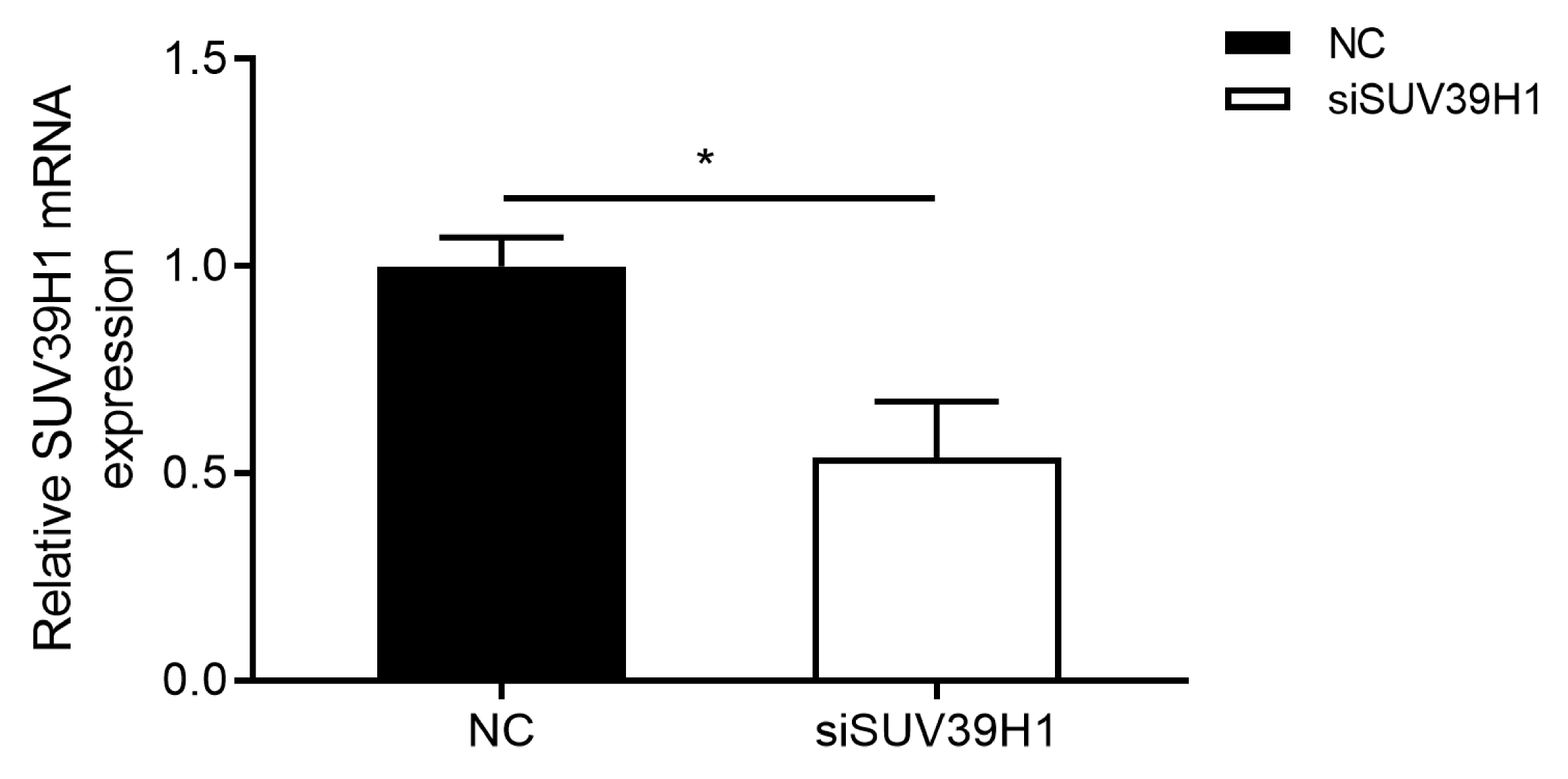

Supplement: Supplementary file 5 — Additional file 5: Fig. S5. SUV39H1 expression decreased with the presence of SUV39H1 siRNA in murine corneal epithelium during corneal epithelial wound healing (CEWH). RT-qPCR was used to analyze the expression levels of SUV39H1 in the negative control (NC) and the SUV39H1 siRNA-injected groups (n = 3/group). [file 40662_2022_275_MOESM5_ESM.tif]

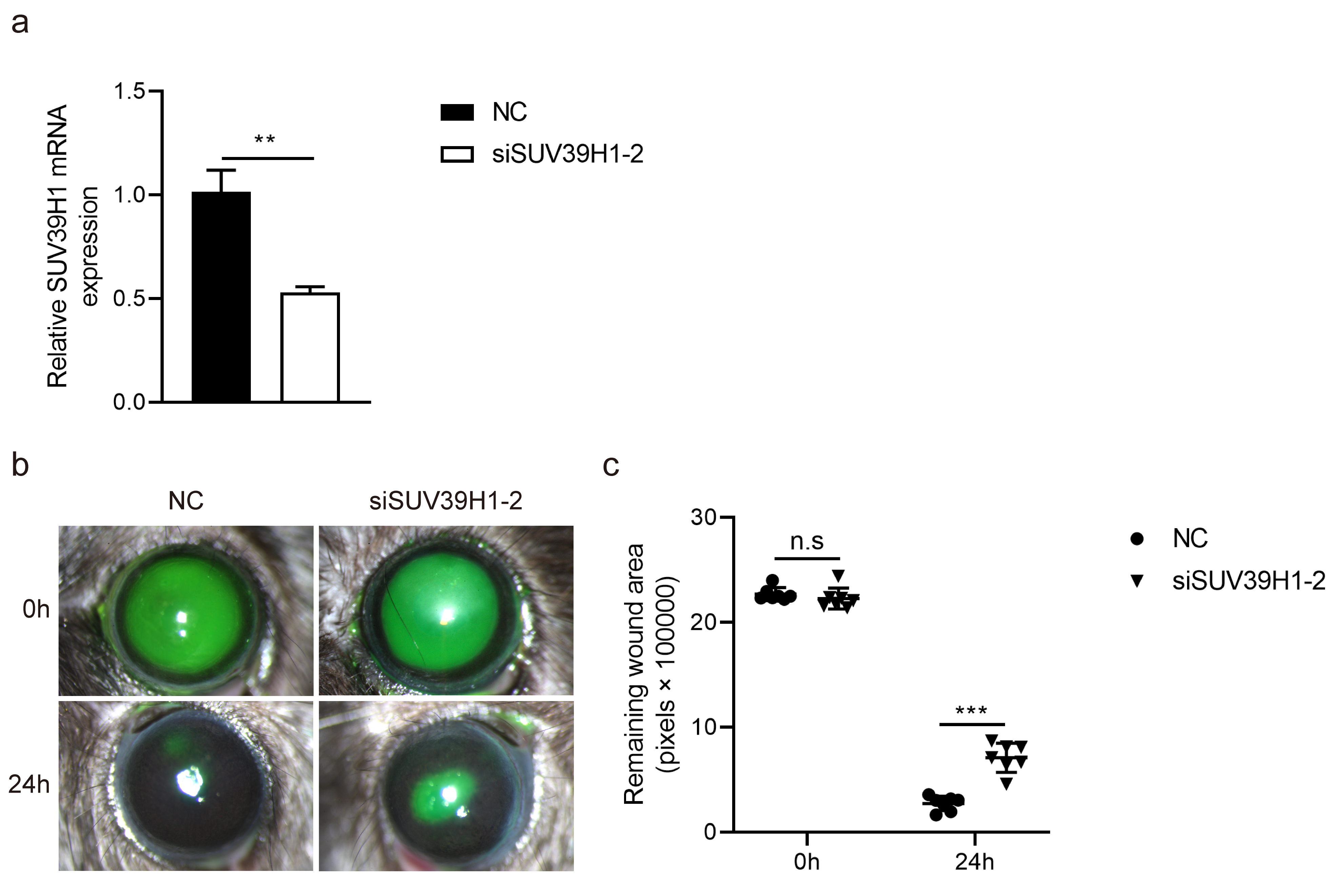

Supplement: Supplementary file 6 — Additional file 6: Fig. S6. SUV39H1 repression with a different siRNA remarkedly delays corneal epithelial wound closure. a The expression level of SUV39H1 in the corneal epitheliums of negative control (NC) and the SUV39H1 siRNA-injected groups were quantified by RT-qPCR (n = 4/group). b Representative images of fluorescein sodium-stained corneas in SUV39H1 siRNA or NC injected groups. c Scatter plots of residual epithelial defects of corneas in SUV39H1 siRNA and NC injected groups (n = 7/group). The remaining wound areas are presented in pixels of size. [file 40662_2022_275_MOESM6_ESM.tif]

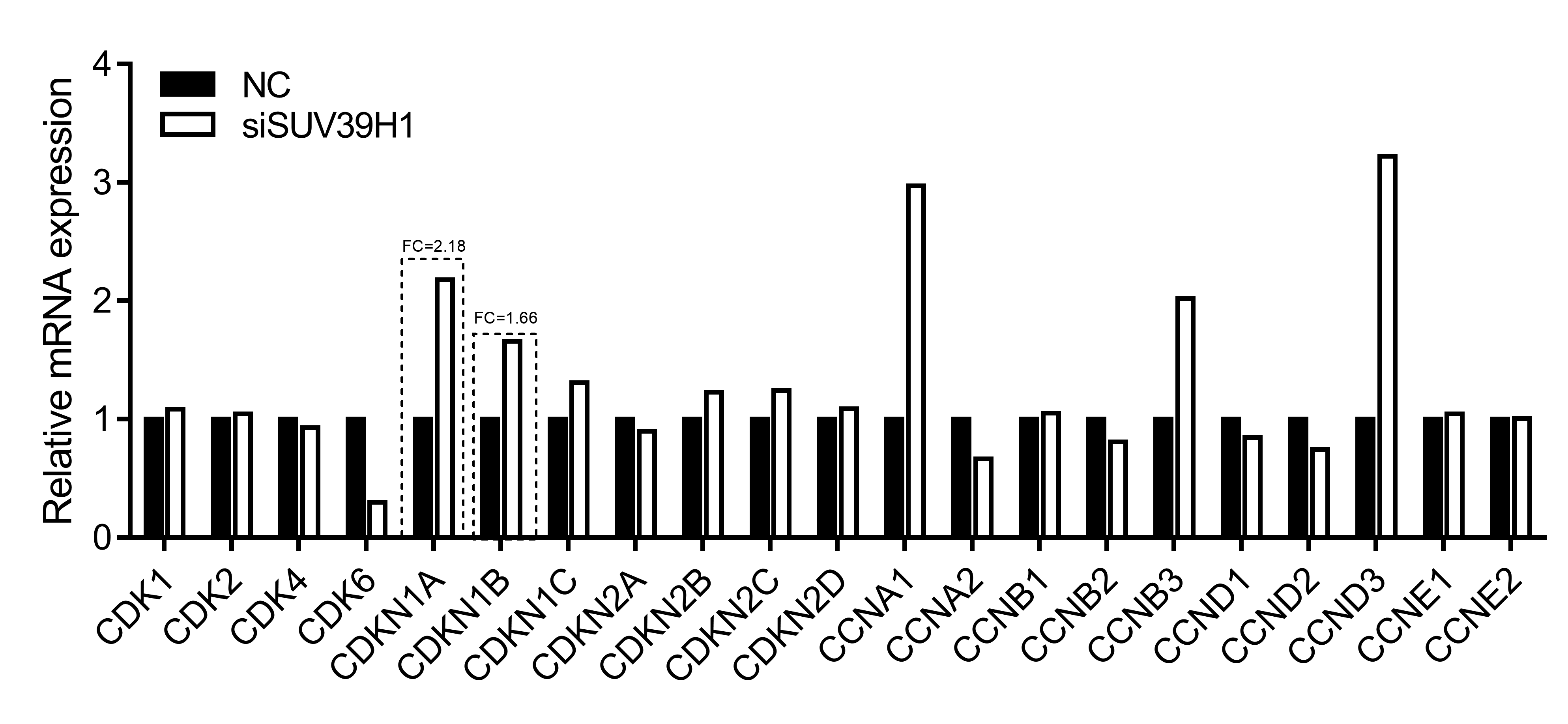

Supplement: Supplementary file 7 — Additional file 7:Fig. S7. Expression of cell cycle associated genes in human corneal epithelial cells (HCECs) transfected with SUV39H1 siRNA. RT-qPCR was used to systematically analyze the alteration of cell cycle associated genes in HCECs at 48 h after transfection with negative control (NC) or SUV39H1 siRNA. [file 40662_2022_275_MOESM7_ESM.tif]

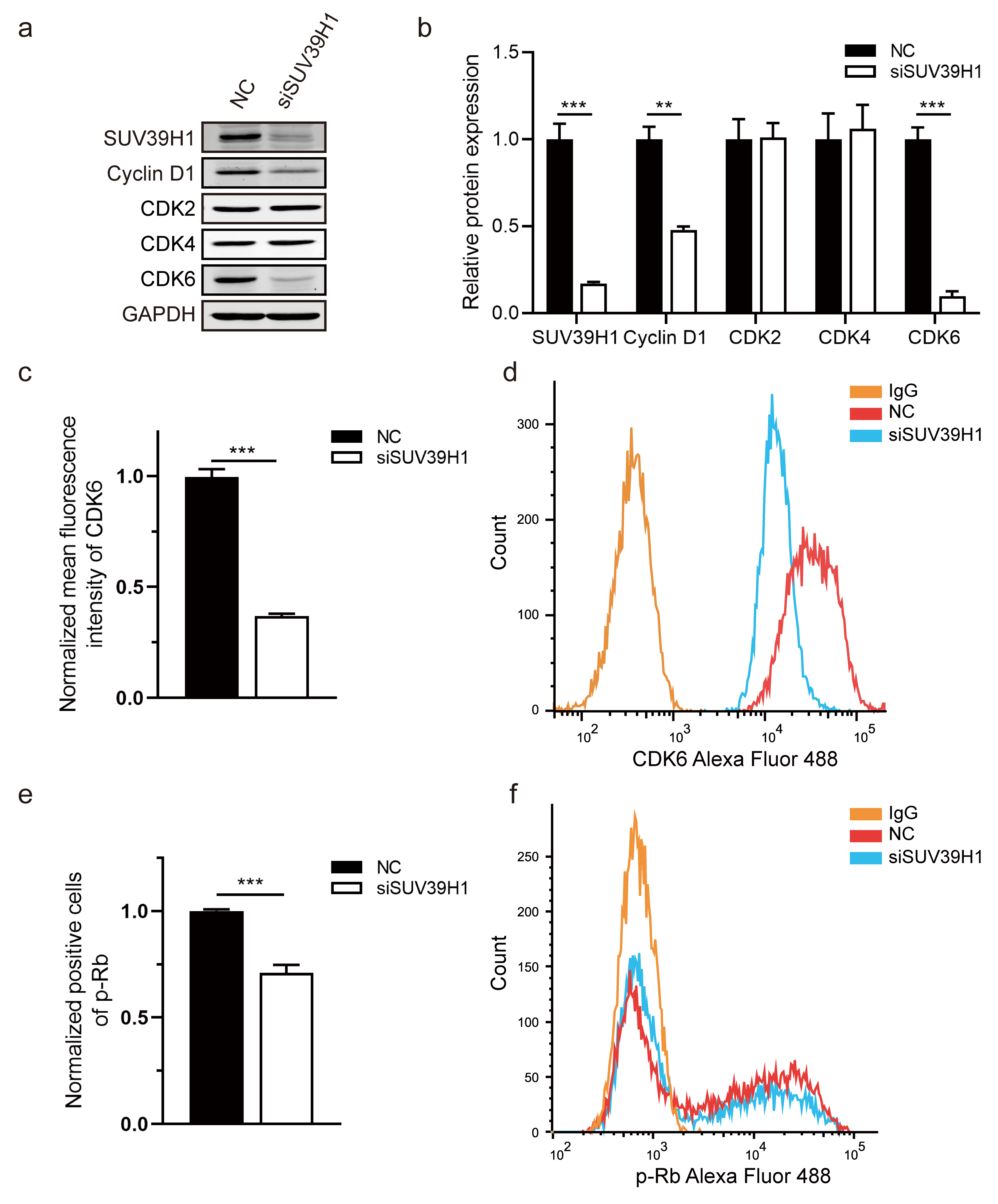

Supplement: Supplementary file 8 — Additional file 8: Fig. S8. SUV39H1 repression significantly decreases the phosphorylation level of Rb, Cyclin D1, and CDK6 in human corneal epithelial cells (HCECs). a Western blotting detected the protein levels of Cyclin D1, CDK2, CDK4, and CDK6 in NC or SUV39H1 siRNA transfected HCECs. b The protein levels of Cyclin D1 and CDKs were quantified from three independent replicates. c Histogram of normalized mean fluorescence intensity of CDK6 in NC siRNA or SUV39H1 siRNA transfected HCECs by fluorescence activated cell sorting (FACS) (n = 3/group). d Representative result of CDK6 in FACS is shown in HCECs after siRNA transfection. e Histogram of normalized positive p-Rb cells in negative control (NC) or SUV39H1 siRNA transfected HCECs (n = 4/group) via FACS. The percentage of positive p-Rb cells in HCECs transfected with NC siRNA acted as the normalized control. f Representative line chart of the positive p-Rb cells in HCECs transfected with NC or SUV39H1 siRNA via FACS. [file 40662_2022_275_MOESM8_ESM.tif]

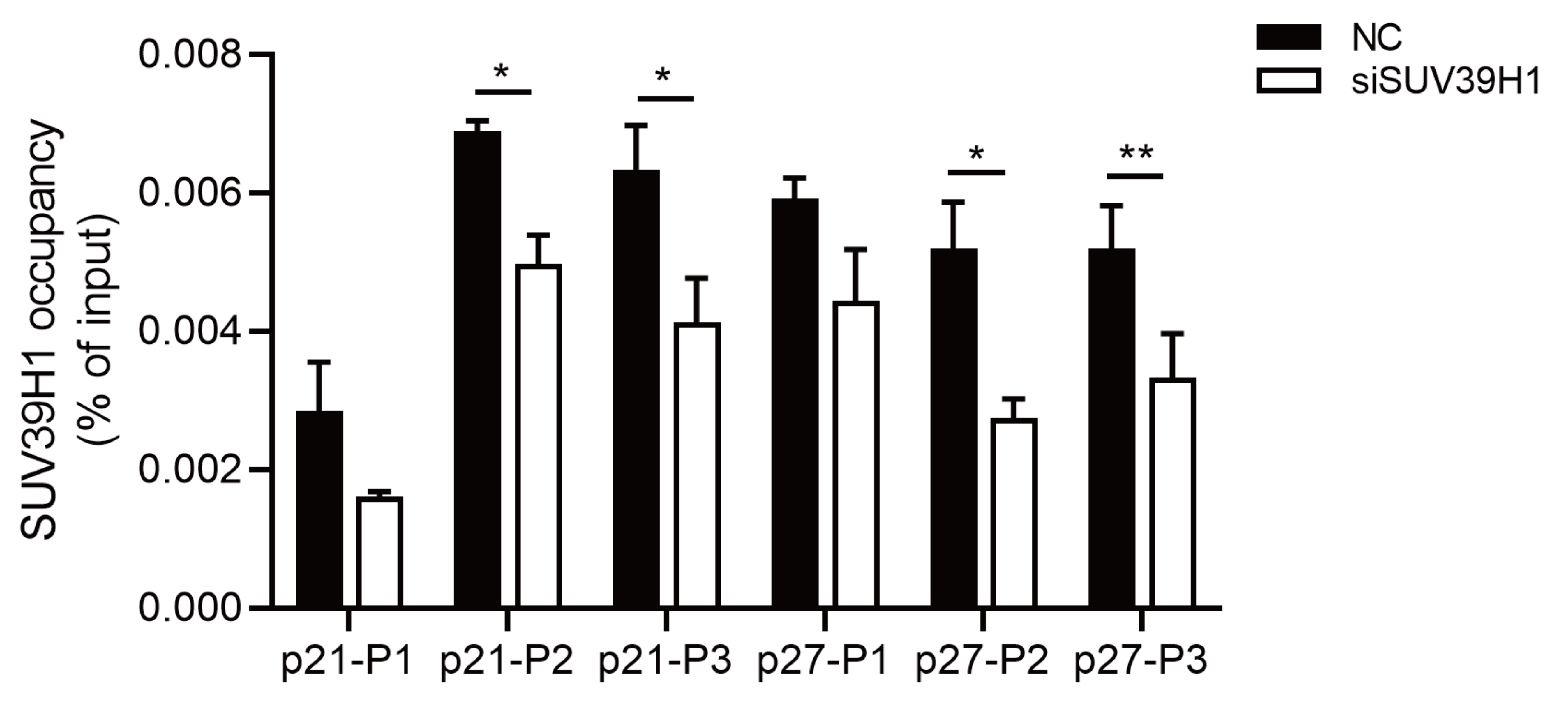

Supplement: Supplementary file 9 — Additional file 9: Fig. S9. SUV39H1 directly binds to the p27 and p21 promoters in human corneal epithelial cells (HCECs). Chromatin immunoprecipitation-quantitative polymerase chain reaction (ChIP-qPCR) was performed to analyze SUV39H1 occupancy at the p27 and p21 promoters at 48 h after siRNA transfection in HCECs (n = 3/group). The percentage (%) of the input signal is used to express the results. [file 40662_2022_275_MOESM9_ESM.tif]
